# The Rosenberg view outperforms conventional AP radiographs in detecting medial knee osteoarthritis: A matched‐pair analysis using intraoperative cartilage status

**DOI:** 10.1002/jeo2.70582

**Published:** 2025-12-07

**Authors:** Clemens Clar, Amir Koutp, Lukas Leitner, Jakob Tettmann, Andreas Leithner, Patrick Sadoghi

**Affiliations:** ^1^ Department of Orthopaedics and Trauma Medical University of Graz Graz Austria; ^2^ Alps Surgery Institute Clinique Generale Annecy Annecy France; ^3^ Department of Orthopaedics and Trauma Surgery Musculoskeletal University Center Munich (MUM), LMU University Hospital Munich Germany

**Keywords:** cartilage, knee osteoarthritis, knee X‐ray, Rosenberg view, total knee arthroplasty

## Abstract

**Purpose:**

This study aimed to compare the diagnostic performance of the Rosenberg view against conventional anteroposterior (AP) radiographs for detecting knee osteoarthritis (OA). Using intraoperative cartilage status as the gold standard, the diagnostic accuracy of both views was evaluated in a matched‐pair analysis. It was hypothesised that the Rosenberg view would demonstrate superior sensitivity in detecting cartilage wear, particularly in the medial compartment.

**Methods:**

This retrospective matched‐pair analysis evaluated 150 knee OA patients undergoing arthroplasty with same‐day preoperative AP and Rosenberg radiographs (≤1 week before surgery). Two blinded observers independently graded medial and lateral compartments using the KL scale (1–4), with interobserver reliability assessed via weighted kappa coefficients. Intraoperative cartilage status (‘worn’/‘unworn’) served as the reference standard. Statistical analysis included Firth's penalised logistic regression (to address complete separation), ROC curve analysis with DeLong's test for AUC comparisons and performance metrics at optimal probability thresholds determined by Youden's index.

**Results:**

The cohort (93 females, 57 males; mean age 65.6 ± 8.8 years; mean BMI 30.2 ± 5.44 kg/m²) demonstrated superior diagnostic performance for Rosenberg views in medial compartment assessment (AUC 0.976, 95% CI 0.96–0.99 vs AP 0.899, *p* = 0.017), with excellent interobserver agreement (weighted *κ* = 0.99). At the optimal threshold (0.41), sensitivity was 62.0% and specificity was 81.0%. Lateral compartment analysis revealed comparable performance between views (Rosenberg AUC 0.756 vs. AP 0.706, *p* = 0.11), though study power was limited (44.4%) for this comparison.

**Conclusions:**

These findings support the consideration of the Rosenberg view in routine diagnostic workflows for knee OA to improve detection of medial compartment disease and better inform treatment planning.

**Level of Evidence:**

Level II, retrospective sub‐analysis of a randomised controlled trial.

**Abbreviations**
AP: anterior‐posterior
AUC: area under the curve
BMI: body mass index
CI: confidence‐interval
KL: Kellgren Lawrence
NPV: negative predictive value
OA: osteoarthritis
OR: odds ratio
PA: posterior‐anterior
PPV: positive predictive value
ROC: receiver operating characteristic
TKA: total knee arthroplasty

## INTRODUCTION

Knee osteoarthritis (OA) is a chronic and degenerative disorder of the knee joint, characterised by hallmark clinical symptoms such as swelling, pain, joint stiffness, and structural alterations of the joint tissues. Approximately 20% of individuals over 45 years develop knee OA during their lifetime, with women being more frequently affected [10, 11, 22]. While mild to moderate OA is primarily managed conservatively, end‐stage OA is commonly treated with total knee arthroplasty (TKA), a surgical intervention associated with high patient satisfaction, as it typically results in pain relief, functional restoration, and improved quality of life [2, 3, 5].

The Kellgren–Lawrence (KL) grading scale has been established as a standard for assessing knee OA and consists of four grades depending on the joint space narrowing, serving as a crucial decision‐making tool for therapeutic options [3, 14]. Due to its high validity and reliability, the KL score has also been adopted by the World Health Organization for use in epidemiological studies [19]. To evaluate this score, a weight‐bearing anterior‐posterior (AP) radiograph of the knee joint in full extension is typically performed. However, this approach is insensitive in KL grades 2–3, often underestimating disease severity, considering that the predominant pattern of degenerative wear is localised to the posterior tibiofemoral compartment in most cases [7, 17, 19]. As an alternative, the Rosenberg view was developed in 1988, a posteroanterior (PA) weight‐bearing radiograph taken at 45° of knee flexion. Compared to conventional AP imaging, the Rosenberg view is expected to result in significantly higher accuracy, sensitivity, and specificity [20]. Despite a growing consensus on its utility, the adoption of the Rosenberg view into routine clinical practice remains inconsistent [8].

Despite a growing consensus on its utility, the adoption of the Rosenberg view into routine clinical practice remains inconsistent [8]. This inconsistency may be attributable to several practical factors. These include the technical challenges faced by radiographers in obtaining correctly positioned images and the view's primary advantage in detecting isolated flexion compartment OA. In many healthcare systems, patients present with advanced disease where both flexion and extension joint spaces are affected, at which point standard AP views may be deemed sufficient for diagnosis. Furthermore, there remains a lack of studies directly comparing these radiographic views against a definitive intraoperative gold standard in a matched‐pair design. Therefore, the aim of this study was to compare the diagnostic performance of conventional AP radiographs and the Rosenberg view, using intraoperative cartilage status as the reference. It was hypothesised that Rosenberg's view of radiographs would demonstrate significantly higher sensitivity, particularly in early stages, where conventional AP views tend to underestimate disease severity.

## MATERIALS AND METHODS

### Study design and participants

This study was a retrospective matched‐pair analysis of prospectively collected data from patients undergoing knee arthroplasty. Preoperative AP and Rosenberg radiographs were obtained for each knee and independently graded by two independent observers using the KL scale (grades 1–4) for the medial and lateral compartments at four‐week intervals. Each view was scored twice to enable assessment of interobserver agreement. Intraoperative cartilage status was recorded in operative reports as ‘worn’ or ‘unworn’ for both compartments, and these findings were used as the reference standard after being recoded into binary variables (1 = worn, 0 = unworn). All necessary data were retrieved from the hospital's local information system, MEDOCS.

### Inclusion and exclusion criteria

Both male and female patients were included, provided that both standard AP and Rosenberg view radiographs were available. The two radiographic examinations had to be on the same day and at a maximum 1 week prior to surgery to ensure comparable baseline conditions. Additionally, radiographs had to meet predefined quality standards. All patients aged 18 years and older were included.

Consequently, all patients with relevant prior knee surgeries and patients who explicitly requested exclusion from the study were excluded. Furthermore, cases with non‐numeric KL grades or missing operative findings were excluded from relevant analyses.

### Data collection

**Figure 1:**
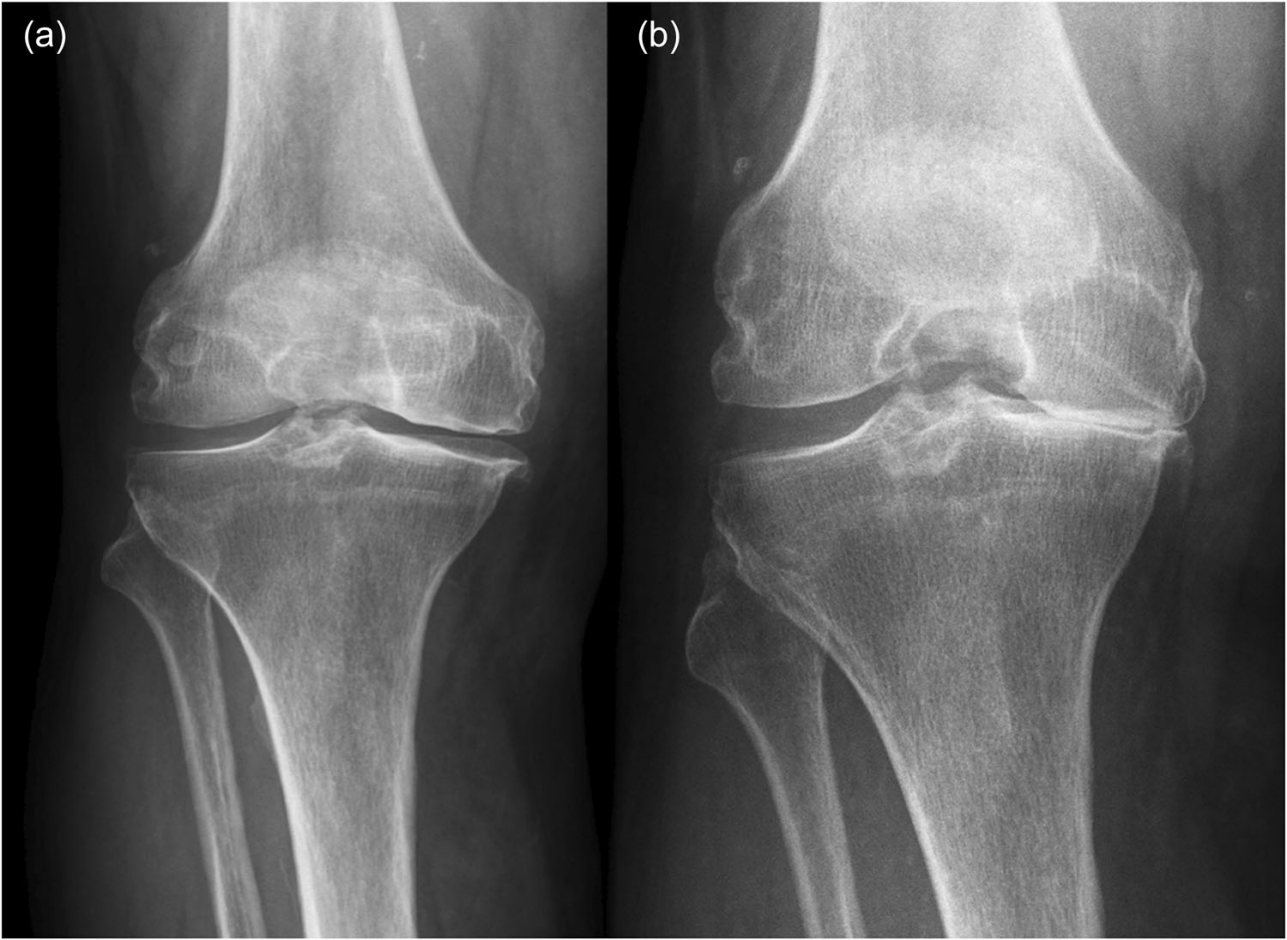
Standard radiographic views used for KL grading: (a) anterior‐posterior (AP) weight‐bearing view and (b) Rosenberg view (45° flexion posteroanterior weight‐bearing view). Both images demonstrate typical positioning for osteoarthritis assessment, with the Rosenberg view providing enhanced visualisation of posterior femoral condyles and tibiofemoral joint spaces.

The data collection was carried out from March 2021 to November 2024 by orthopaedic and trauma surgeons. All relevant data from the hospital documentation system were carefully reviewed. Radiographic assessment was performed using standardised AP and Rosenberg view (Figure 1). Two blinded observers (C.C. and J.T.) independently assessed all images according to the Kellgren–Lawrence grading system, focusing on joint space narrowing, osteophytes, and subchondral sclerosis. Interobserver reliability was calculated, and discrepancies were resolved through consensus. Demographic variables included age, gender, height, and weight, with body mass index (BMI) calculated as weight in kilograms divided by height in metres squared.

### Outcomes

The primary outcomes of this study were the classification of the AP and Rosenberg view radiographs according to the KL grading system in four grades, with grade 1 representing the mildest form of OA and grade 4 representing the most advanced. Consequently, the results of the two radiographic modalities were compared. Additionally, demographic data were collected and analysed.

### Statistical analysis

Descriptive statistics were calculated for patient demographics and KL grade frequencies. Interobserver agreement for the ordinal KL grades was assessed using a quadratically weighted kappa coefficient.

To evaluate the predictive performance of each radiographic view, univariable and multivariable logistic regression models were developed. Due to the presence of quasi‐complete separation in the data, Firth's penalised logistic regression was used for all models to ensure stable and unbiased estimates. Kellgren‐Lawrence grades were treated as a categorical variable to derive odds ratios (ORs) with 95% confidence intervals (CIs) for each grade relative to grade 1. The multivariable models were adjusted for patient gender and BMI.

Model discrimination was assessed using the area under the receiver operating characteristic (ROC) curve. To formally compare the diagnostic accuracy, the AUCs of the extended multivariable models for the AP and Rosenberg views were compared using DeLong's test for two correlated ROC curves. For each model, detailed performance metrics including accuracy, sensitivity, specificity, positive predictive value (PPV), and negative predictive value (NPV) were calculated at the optimal threshold determined by the Youden index.

A post‐hoc power analysis was conducted to determine the statistical power achieved to detect the observed difference in AUCs between the two models. All analyses were performed separately for the medial and lateral compartments using R version 4.4.1 (R Foundation for Statistical Computing, Vienna, Austria).

## RESULTS

The cohort comprised 150 knee OA cases from 93 (62%) female and 57 (38%) male patients with a mean age of 65.6 ± 8.8 years. The BMI ranged from 20.3 to 50.1 kg/m², with a mean of 30.2 ± 5.44 kg/m². In the medial compartment, AP grades were most frequently grade 3 (*n* = 74, 49.3%) or grade 2 (*n* = 60, 40.0%), while Rosenberg grades were most frequently grade 4 (*n* = 113, 75.3%). In the lateral compartment, AP grades were most frequently grade 2 (*n* = 78, 52.0%) or grade 1 (*n* = 47, 31.3%), and Rosenberg grades were most frequently grade 2 (*n* = 63, 42.0%) or grade 4 (*n* = 32, 21.3%). Baseline demographic and radiographic characteristics of the study population are summarised in Table 1.

**Table 1:** Baseline demographic and radiographic characteristics.

Characteristic	Total cohort (*n* = 150)	Medial compartment KL grades	Lateral compartment KL grades
Age (years)	65.6 ± 8.8	–	–
Sex			
Female	93 (62.0%)	–	–
Male	57 (38.0%)	–	–
BMI (kg/m²)	30.2 ± 5.44	–	–
AP view grades			
Grade 1	–	12 (8.0%)	47 (31.3%)
Grade 2	–	60 (40.0%)	78 (52.0%)
Grade 3	–	74 (49.3%)	19 (12.7%)
Grade 4	–	4 (2.7%)	6 (4.0%)
Rosenberg grades			
Grade 1	–	0 (0.0%)	15 (10.0%)
Grade 2	–	10 (6.7%)	63 (42.0%)
Grade 3	–	27 (18.0%)	40 (26.7%)
Grade 4	–	113 (75.3%)	32 (21.3%)

*Note*: Values are presented as mean ± standard deviation or number (percentage). Grades refer to the severity of osteoarthritis as evaluated on AP and Rosenberg radiographic views for the medial and lateral compartments, respectively.

Abbreviations: AP, anteroposterior; BMI, body mass index; KL, Kellgren–Lawrence.

Comparative model performance across compartments is detailed in Table 2.

**Table 2:** Comparison of radiographic view performance metrics by compartment.

	Medial compartment		Lateral compartment	
	Rosenberg view	AP view	Rosenberg view	AP view
AUC (95% CI)	0.976 (0.96–0.99)	0.899 (0.86–0.94)	0.756 (0.68–0.83)	0.706 (0.63–0.78)
Optimal threshold	0.41	0.47	0.45	0.49
Sensitivity	62.0%	68.3%	76.1%	81.7%
Specificity	81.0%	72.7%	57.0%	49.4%
PPV	96.5%	95.1%	76.9%	71.4%
NPV	75.0%	66.7%	68.4%	63.9%
Power	94.9%		44.4%	

*Note*: Values represent diagnostic performance of the Rosenberg and anteroposterior (AP) radiographic views in evaluating medial and lateral compartments.

Power indicates the statistical power of each comparison.

Abbreviations: AUC, area under the receiver operating characteristic curve; CI, confidence interval; NPV, negative predictive value; PPV, positive predictive value.

For the medial compartment, interobserver agreement was excellent, with weighted kappa values of 0.94 (95% CI: 0.90–0.98) for AP views and 0.99 (95% CI: 0.98–1.00) for Rosenberg views. Firth's penalised logistic regression revealed strong associations between Rosenberg KL grade and intraoperative wear (OR = 75.3, 95% CI: 7.6–2589.8, *p* < 0.001). However, the extremely wide confidence interval indicates significant uncertainty in the magnitude of this estimate. This statistical artefact is likely a result of the strong discrimination combined with limited variation in the outcome within this surgical cohort, where a high proportion of cases (75.3%) were graded KL 4 on Rosenberg views, leading to quasi‐complete separation in the data.

**Figure 2:**
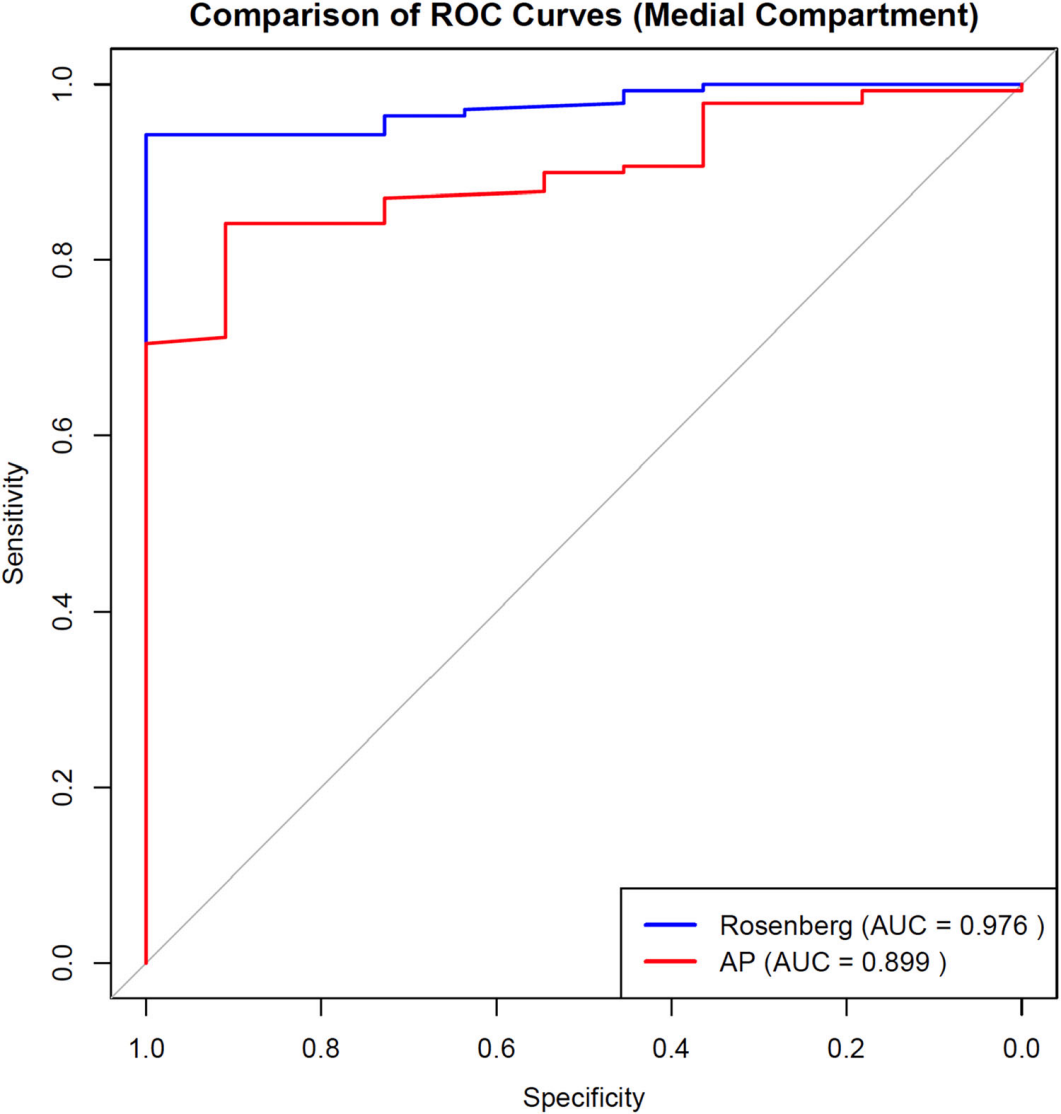
Receiver operating characteristic (ROC) curves comparing the predictive performance of Rosenberg and AP views for detecting intraoperative cartilage damage in the medial compartment. Receiver operating characteristic (ROC) curves comparing Rosenberg and AP views for detecting intraoperative cartilage damage. Medial compartment: Rosenberg AUC = 0.976 (optimal cutoff = 0.41) vs. AP AUC = 0.899 (DeLong's *p* = 0.017).

As shown in Table 2, the extended model including age, BMI and gender demonstrated excellent discrimination (AUC = 0.976, 95% CI: 0.96–0.99), significantly outperforming the AP view model (AUC = 0.899; DeLong's test *p* = 0.017) (Figure 2). At the optimal probability threshold, the Rosenberg model achieved a sensitivity of 62.0% and specificity of 81.0%. The Rosenberg view's high specificity (81.0%) suggests value in ruling in medial compartment damage, while its moderate sensitivity (62.0%) may limit use as a standalone screening tool. The post‐hoc power analysis confirmed adequate power (94.9%) to detect this difference.

**Figure 3:**
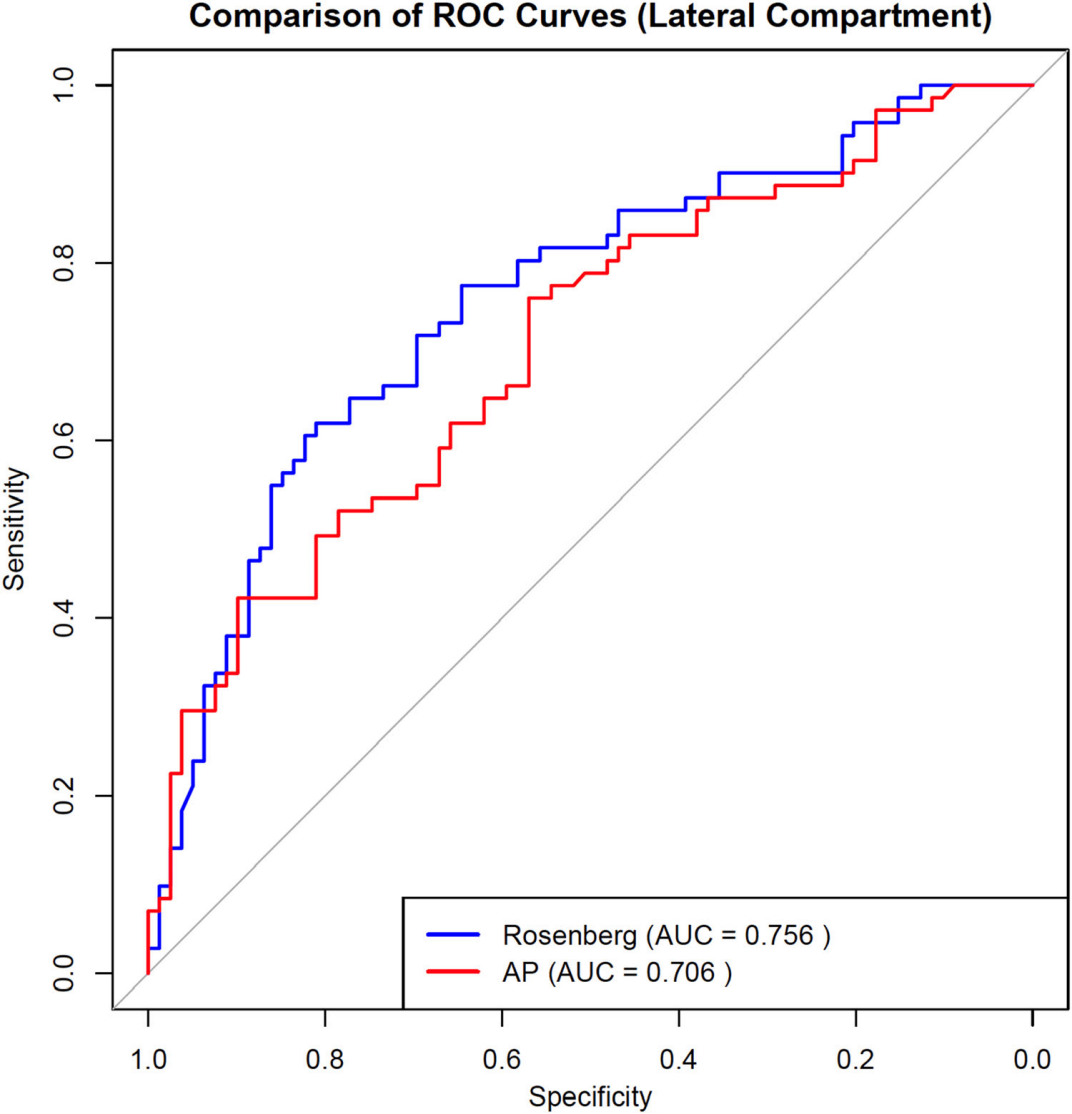
Receiver operating characteristic (ROC) curves comparing the predictive performance of Rosenberg and AP views for detecting intraoperative cartilage damage in the lateral compartment. Receiver operating characteristic (ROC) curves comparing Rosenberg and AP views for detecting intraoperative cartilage damage. Lateral compartment: Rosenberg AUC = 0.756 (optimal cutoff = 0.45) vs. AP AUC = 0.706 (*p* = 0.11). Dashed lines indicate optimal thresholds maximising Youden's index.

For the lateral compartment (Table 2), interobserver agreement remained strong, with weighted kappa values of 0.90 (95% CI: 0.85–0.95) for AP and 0.93 (95% CI: 0.89–0.97) for Rosenberg views. The Rosenberg model showed significant predictive ability (OR = 2.03, 95% CI: 1.25–2.89, *p* < 0.001) with fair discrimination (AUC = 0.756, 95% CI: 0.68–0.83), while the AP model demonstrated similar performance (AUC = 0.706, 95% CI: 0.63–0.78; DeLong's test *p* = 0.11) (Figure 3). At the optimal threshold, the Rosenberg model achieved 76.1% sensitivity and 57.0% specificity. The post‐hoc power was limited (44.4%), suggesting the analysis may have been underpowered to detect small differences.

## DISCUSSION

This study confirmed the hypothesis that the Rosenberg view provides superior diagnostic performance for detecting medial compartment cartilage degeneration compared to conventional AP radiographs, using intraoperative findings as the reference standard. The analysis demonstrated a significantly higher area under the ROC curve (AUC) for the Rosenberg view in the medial compartment (0.976 vs. 0.899, *p* = 0.017). This finding supports its use for more accurate disease staging, which is critical for appropriate treatment planning, particularly in cases where standard AP views may underestimate the extent of posterior cartilage wear.

Considering the predicted increase in the prevalence of knee OA, it is crucial to have a diagnostic method that is both cost‐effective and highly accurate [9, 24].

While magnetic resonance imaging (MRI) is often considered the most detailed imaging modality for joint pathology, its role in the routine diagnosis of knee OA warrants careful consideration. As highlighted in a recent scoping review by Salamah et al. [21], MRI demonstrates moderate sensitivity (61%–74%) and high specificity (92%–95%) for symptomatic knee OA. However, its high cost, limited accessibility, and potential for identifying incidental findings that may not correlate with the patient's symptoms make it less practical for initial or widespread screening. In a prospective study, Mortensen et al. [18] concluded that even in end‐stage OA, the Rosenberg view achieves a detection rate comparable to MRI for cartilage height, suggesting it is a more pragmatic standard imaging modality. Our findings reinforce this perspective, demonstrating that the Rosenberg view offers excellent diagnostic performance at a fraction of the cost, making its integration into routine diagnostic algorithms a clearly warranted and efficient strategy.

It is important to note that the medial compartment of the knee is the most frequently affected site, with isolated medial knee osteoarthritis accounting for up to 50% of cases according to current evidence, which aligns with the findings of the present study [6, 12, 23]. For this compartment, the Rosenberg view demonstrated a high positive predictive value (PPV) of 96.5% and a high specificity of 81.0%, indicating it is highly effective at correctly identifying the presence of cartilage wear. However, its moderate sensitivity (62.0%) suggests that a negative finding on a Rosenberg view does not definitively rule out disease, highlighting that it should be interpreted within the broader clinical context rather than as a standalone screening tool [1, 13].

In contrast, this study did not demonstrate a significant improvement over conventional AP radiographs for the detection of lateral knee osteoarthritis, despite very high interobserver agreement and satisfactory AUC values. The analysis of the lateral compartment was underpowered, which may have limited the ability to detect a statistically significant difference. Considering that posterior femoral condyle wear is frequently observed in the lateral compartment, current evidence in diagnostic practice continues to favour the use of Rosenberg view radiographs [4, 15, 16].

### Limitations

This study has several limitations. First, it was conducted as a retrospective, single‐centre investigation. The patient selection process may have introduced selection bias, as only cases with complete radiographic datasets for both views and documented intraoperative findings were included, while patients with relevant prior knee surgeries were excluded. The intraoperative assessment of cartilage status, which served as the reference standard, was based on a binary classification (‘worn’ vs. ‘unworn’), thereby only capturing rough gradations in cartilage damage. Additionally, while the medial compartment analysis was well‐powered (94.9%), the lateral compartment comparison had limited power (44.4%) to detect smaller differences. The absence of long‐term radiographic follow‐up precludes definitive conclusions regarding the durability of the results, and the single‐centre design may limit the generalisability of the findings.

## CONCLUSION

The findings of this study strongly suggest that the Rosenberg view enhances the diagnostic accuracy of radiographic OA assessment, particularly for medial knee osteoarthritis. It is recommended that the Rosenberg view be strongly considered for incorporation into routine diagnostics to minimise underestimation of disease severity and optimise treatment planning.

## AUTHOR CONTRIBUTIONS

**Clemens Clar**: Conceptualisation; methodology; analysis of data; investigation; data curation; writing—original draft preparation. **Amir Koutp**: Conceptualisation; methodology; analysis of data; investigation; data curation; supervision; writing—review and editing. **Lukas Leitner**: data curation; supervision; writing—review and editing. **Andreas Leithner**: Conceptualisation; methodology; analysis of data; investigation; data curation. **Jakob Tettmann**: Conceptualisation; methodology; analysis of data; investigation; data curation; supervision; writing—review and editing. **Patrick Sadoghi**: Conceptualisation; methodology; analysis of data; investigation; data curation; supervision; writing—review and editing.

## CONFLICT OF INTEREST STATEMENT

Andreas Leithner received Industry grants from DePuy Synthes, Johnson & Johnson, alphamed and Medacta. Patrick Sadoghi received Industry grants from DePuy Synthes, Johnson & Johnson, alphamed and Medacta; Editorial Board Member for JOA, KSSTA and Arthroscopy. The remaining authors declare no conflicts of interest.

## ETHICS STATEMENT

Approved by the Ethics Committee of the Medical University of Graz (EK: 30‐253 ex 22/23). All patients provided informed consent.

## Data Availability

Data are available from the corresponding author upon reasonable request.
